# A Study of Changes in Newly Graduated Registered Nurses’ Resilience, Satisfaction With the Work Environment, and Intention to Stay During a Two‐Year Training Program and Corresponding Mediating Effects: A Longitudinal Study

**DOI:** 10.1155/jonm/9633853

**Published:** 2026-05-10

**Authors:** I-Shiang Tzeng, Chu-Hsuan Cheng, Ru-Wen Liao

**Affiliations:** ^1^ Department of Research, Taipei Tzu Chi Hospital, Buddhist Tzu Chi Medical Foundation, New Taipei City, Taiwan, tzuchi.com.tw; ^2^ Department of Nursing, Taipei Tzu Chi Hospital, Buddhist Tzu Chi Medical Foundation, New Taipei City, Taiwan, tzuchi.com.tw; ^3^ School of Nursing, Tzu Chi University, Hualien, Taiwan, tcu.edu.tw

## Abstract

**Background:**

Newly graduated registered nurses (NGRNs) are likely to leave their jobs because they cannot adapt to nursing work. In Taiwan, a two‐year on‐the‐job training program was implemented to help NGRNs meet the demands and adapt to nursing jobs. However, the effects of the program on NGRNs’ satisfaction with the work environment, individual resilience, and intention to stay, as well as the relationships among these factors, remain unclear.

**Aim:**

The aim of this study was to investigate the changes in NGRNs’ perceptions of the work environment, individual resilience, and intention to stay after they complete each stage of the on‐the‐job training program and to examine the mediating effect of resilience on the relationship between the work environment and nurses’ intention to stay.

**Methods:**

A repeated‐measures study was conducted at a medical center in Taiwan to survey NGRNs after they completed each stage of the training program. The survey questionnaire included the NGRNs’ background information, satisfaction with the work environment, individual resilience, and intention to stay. Changes across the training stages and the associated mediating effects were analyzed.

**Results:**

We observed that NGRNs’ satisfaction with the work environment (*p* < 0.001) and intention to stay (*p* < 0.001) decreased significantly across the three waves of the survey, whereas individual resilience increased significantly (*p* < 0.05). Furthermore, we observed that individual resilience significantly mediated the impact of satisfaction with the work environment on nurses’ intention to stay (*p* < 0.05).

**Conclusion:**

The training program can significantly improve NGRNs’ individual resilience. However, with respect to their satisfaction with the work environment and intention to stay, the training may require adjustment; alternatively, additional measures may be needed to mitigate the effects of job changes on NGRNs.

## 1. Introduction

### 1.1. Background

In recent years, both the nursing shortage and the aging workforce have become urgent global issues [1–3]. In Taiwan, the nursing workforce must expand by 55,000 to 74,000 nurses by 2030 to meet the projected demand [3]. The task of addressing these issues requires a significant influx of newly trained nursing professionals. As such, newly graduated registered nurses (NGRNs) represent a critical resource in efforts to address these two challenges.

Nursing is among the most stressful occupations [5, 6]. In addition, NGRNs require further training and practice after entering employment to meet the demands of clinical practice. They must also adapt to their work environment and the high‐stress nature of their job responsibilities [7–9]. The term “transition shock” has been used to describe this experience among NGRNs, as they begin to independently assume full professional responsibility in comprehensive patient care, a role characterized by considerable stress [7]. If NGRNs are unable to cope with these situations, they may ultimately consider leaving their job [8, 9].

In Taiwan, hospitals are required to implement a two‐year on‐the‐job training program for NGRNs, also known as the Nurse Post‐Graduate Year (NPGY) training program, as mandated by the Ministry of Health and Welfare in 2007 [4]. This program is divided into three stages and aims to equip NGRNs with essential nursing skills and practical experience. With respect to this training program, the first stage is completed 3 months after onboarding, the second stage is completed after 1 year, and the final stage is completed at the end of the second year. Within each stage, NGRNs are assigned work responsibilities that correspond to their competency levels. During the **first stage** (the basic curriculum stage), the training program is designed to enable NGRNs to perform basic nursing care and practices and to cope with work‐related stress. In this stage, NGRNs provide care to 6–8 patients under the supervision and hands‐on teaching of preceptors. During the **second stage** (the core curriculum stage), the training program is designed to enable NGRNs to perform general nursing practices, such as administrative duties and interdisciplinary collaboration. At this point, they can independently care for 7–11 patients and are responsible for conducting basic shift handovers while under the supervision of the preceptor, nurse manager, or care team leader. During the **final stage** (the professional course stage), the training program is designed to enable NGRNs to perform advanced and specialized nursing care, such as providing care according to their unit specialty and managing patients with severe or complex conditions. At this point, they can independently care for 7–11 patients, including those with severe or complex conditions, and conduct interdisciplinary shift handovers while under the supervision of preceptors, nurse managers, or care team leaders. More detailed information about the program is provided in Supporting Information 1. Through this training program, the government and hospitals aim to strengthen NGRNs’ ability to meet the demands of clinical practice, thereby improving individual resilience, mitigating the impact of transition shock, and ultimately increasing the intention to stay [10].

### 1.2. Theoretical Framework

In this study, the variables of interest included satisfaction with the work environment, individual resilience, and turnover intention/intention to stay. Hartmann et al. [11] suggested that integrated resource theories provide a comprehensive theoretical framework for explaining the role of individual resilience in work‐related outcomes by combining job demands–resources (JD–R) theory and conservation of resources (COR) theory.

**FIGURE 1:**
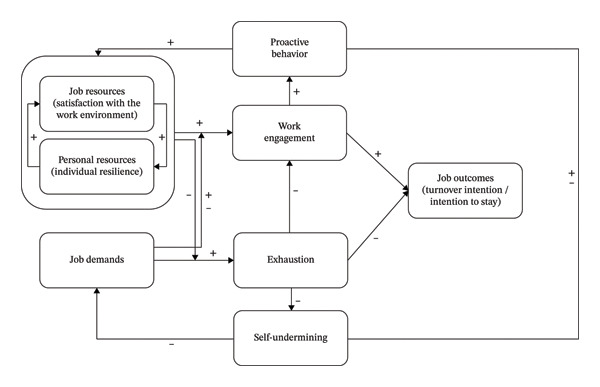
The job demands–resources model. *Note.* Adapted and redrawn from Bakker and Demerouti [78].

The theoretical framework of the JD–R model was initially proposed by Demerouti et al. [12], as shown in Figure 1. According to this framework, job conditions can be broadly classified into job demands and job resources. Job demands refer to aspects of the job that require sustained physical and/or psychological effort and are therefore associated with certain physiological and/or psychological costs. In contrast, job resources refer to aspects of the job that motivate individuals, help them achieve work goals, buffer the impact of job demands, or promote learning and personal growth [13]. Increased job demands are associated with increased levels of exhaustion, which in turn negatively affect job outcomes. In contrast, increased job resources increase employees’ work engagement, which has a positive effect on job outcomes [13]. Although job performance is often presented as a representative outcome in the original JD–R model, the framework also encompasses other work‐related outcomes, including turnover intention [13, 14]. Personal resources refer to individuals’ positive self‐evaluations of their ability to control and successfully influence their environment, such as optimism, self‐efficacy, and resilience; these resources play a mediating role in the relationship between job resources and work engagement and contribute to the accumulation of further job resources [13, 15, 16].

The theoretical framework of COR theory was proposed by Hobfoll [17]. It posits that individuals strive to acquire and protect resources that are central to them, that stress occurs when these resources are threatened by loss, actually lost, or when efforts to acquire these resources fail, and that individuals may intentionally engage in behaviors to prevent or minimize resource loss and to gain additional resources to reduce the likelihood that core elements will be harmed. On the basis of this perspective, individuals’ actions are guided by four core principles. First, individuals place greater emphasis on resource loss than on resource gain. Second, individuals must invest in existing resources to gain additional resources. Third, when individuals experience resource loss, the value of resource gain increases. Finally, when personal resources are excessively depleted and can no longer protect core elements, individuals may enter a protective mode in which their behavior becomes more defensive or aggressive and may even involve irrational actions [18]. Within this framework, resources can be categorized into different types, including personal, material, energy, and condition resources [17, 18]. These resources function to protect core elements, which are the things that individuals centrally value, such as health, well‐being, family, self‐esteem, and a sense of purpose and meaning in life [18]. In addition, the theory includes the principles of resource caravans and resource caravan passageways, which emphasize that resources do not exist in isolation but travel in packs or caravans and that they exist within individuals’ ecological environments, which may either foster and nurture or limit and block resource creation and sustenance [18].

These two theoretical frameworks can be integrated to provide a more comprehensive explanation of how work influences individuals and their work‐related outcomes. JD–R theory provides a theoretical framework describing how job demands and job resources influence employees and their work outcomes, whereas COR theory explains the principles and mechanisms through which changes in resources influence individuals’ intentions and behaviors. Within this integrated perspective, job resources facilitate the development of personal resources, and these resources help individuals counteract resource loss, such as by gaining additional resources or engaging in actions to mitigate resource loss, thereby influencing individuals’ behavioral responses and work‐related outcomes. More specifically, within this integrated theoretical framework, job resources promote the development of personal resources, and personal resources mediate the relationship between job resources and work‐related outcomes.

With respect to the variables of interest, satisfaction with the work environment reflects the extent to which an organization and its resources provide nurses with effective support in the context of professional practice [19–21]. Cox et al. [19] conceptualized the nursing work environment through characteristics such as manager support, peer support, unit support, workload, and the intention to stay, a framework that has been widely applied in nursing research [22]. These characteristics represent aspects that nurses consider important for their professional practice [22]. Specifically, manager support, peer support, and unit support reflect organizational and social job resources, whereas workload reflects nurses’ perceived adequacy of staffing and the manageability of their workload. Together, these characteristics correspond to job resources within the JD–R model [13] and can also be conceptualized as a constellation of job‐related resources and passageways (i.e., resource caravans and passageways) within the COR framework [23].

Individual resilience refers to a personal capacity or process that enables individuals to adapt in response to adversity. Individuals who exhibit higher levels of resilience tend to respond more positively to stressors and are likely to experience fewer and less severe negative effects after encountering adversity [11, 24]. Individual resilience is not a stable trait; it may be influenced by various factors and may be enhanced through interventions [11, 24, 25]. Hartmann et al. [11] reported that individuals who possess the knowledge, skills, and self‐efficacy to handle workplace situations and tasks can be seen as having the personal resource antecedents to individual resilience. The work environment is another factor that can affect an individual’s resilience and is considered an external factor [26]. Hartmann et al. [11] identified the work environment as a demand‐related antecedent of nurses’ individual resilience, suggested that a more supportive and satisfying work environment is associated with higher levels of individual resilience, and proposed that individual resilience may act as a mediator influencing work‐related outcomes such as turnover intention. This variable is also considered a personal resource within the JD–R model and is consistent with the characteristics of personal resources (i.e., a personal capacity), which are developed through job resources and influence work‐related outcomes [13, 27, 28]. From a COR perspective, however, it is conceptualized as a capacity driven by personal resources that enables individuals to gain resources and to buffer against or recover from resource loss [18, 23, 29].

Taken together, within the integrated theoretical framework, personal resources, operationalized in this study as individual resilience, are developed through job resources, operationalized as satisfaction with the work environment, and mediate the relationship between job resources and work outcomes, namely, turnover intention/intention to stay (Figure 1). The two‐year on‐the‐job training program is designed to provide NGRNs with lower job demands (i.e., reduced workload) and higher job resources (i.e., a more satisfying work environment, such as greater support from senior nurses and structured training courses) immediately after onboarding. These conditions are intended to foster personal resources such as individual resilience, allowing NGRNs to sufficiently develop personal resources before they are required to cope with typical job demands, thereby reducing the likelihood of adverse job outcomes such as high turnover intention and actual turnover during this period. However, as the program progresses, job demands gradually increase to typical clinical levels, while job resources decrease to typical levels. Under these circumstances, according to the integrated model, NGRNs’ individual resilience may be adversely affected by reductions in job resources. Consequently, NGRNs may perceive a threat to their core resources, which may in turn lead to an increase in turnover intention as a strategy to mitigate perceived resource loss, as proposed by the COR model.

### 1.3. Literature Review

#### 1.3.1. Relationships Among Work Environment Satisfaction, Individual Resilience, and Job Outcomes

Several studies have shown that higher levels of satisfaction with the work environment and greater levels of individual resilience are associated with better job outcomes. With respect to satisfaction with the work environment, studies have reported that nurses with high levels of satisfaction with the work environment tend to have higher job satisfaction [21, 30–33], fewer work‐related psychological problems, including burnout [21, 34–36], better work performance [21, 37], stronger intention to stay [21, 30, 33, 35], and lower turnover intention [36]. With respect to individual resilience, studies have reported that nurses with higher levels of resilience tend to have lower levels of mental health problems [38, 39] and burnout [34, 36, 38] as well as lower turnover intention [30, 40] and turnover rates [36, 41].

Studies have also reported that satisfaction with the work environment is associated with individual resilience. Abdulmohdi [34] surveyed 111 staff nurses in the United Kingdom during the second wave of the COVID‐19 pandemic and reported that perceived social support and perceived organizational support were moderately positively correlated with individual resilience. These two factors are key components of satisfaction with the work environment. Yu and Lee [36] surveyed 371 NGRNs in general hospitals in South Korea and reported that satisfaction with the work environment was moderately positively correlated with individual resilience. They also used structural equation modeling to show that individual resilience partially mediated the relationship between satisfaction with the work environment and turnover intention.

#### 1.3.2. Outcomes of the Two‐Year On‐The‐Job Training Program

Studies have shown that NGRNs who participated in the program demonstrated significantly improved nursing abilities [42, 43]. With respect to turnover intention and turnover rate (or, conversely, intention to stay and retention rate), Chen et al. [44] reported that the 1‐year retention rate increased from 73.7% to 90.6% compared with the period before program implementation. However, Su et al. [45] followed newly employed nurses at 1, 3, and 6 months after employment and reported that their turnover intention increased significantly during the sixth month. With respect to individual resilience and satisfaction with the work environment, Liu et al. [46] surveyed nursing students at 3, 6, and 12 months after they began working in hospitals and reported that individual resilience and satisfaction with the work environment remained stable, whereas Su et al. [45] reported that NGRNs with higher levels of individual resilience had lower turnover rates. Cross‐sectional studies have further examined the relationships among these characteristics. Lin et al. [42] reported that NGRNs’ individual resilience affected turnover intention, and Lee et al. [47] reported that individual resilience mediated the effect of the quality of work life on turnover intention among NGRNs who had completed the first stage of the training program. Overall, the literature suggests that NGRNs’ satisfaction with the work environment may remain stable, whereas their intention to stay may decrease during the first year of the program. Their individual resilience appears to be positively associated with the intention to stay (or inversely associated with turnover); however, changes in resilience and in these characteristics over the full two‐year period, as well as the way these characteristics interrelate throughout the program, remain unclear.

## 2. Aims and Objectives

Because previous studies have not examined changes in NGRNs’ satisfaction with the work environment, individual resilience, and turnover intention/intention to stay across the entire two‐year on‐the‐job training program and because most existing studies have investigated the relationships among these characteristics using cross‐sectional designs, this study had two aims.

First, we aimed to investigate changes in NGRNs’ satisfaction with the work environment, resilience, and intention to stay after the completion of each stage of the two‐year on‐the‐job training program.

Second, we aimed to examine whether the relationships among these three characteristics, as proposed in previous cross‐sectional studies, are also applicable to NGRNs when they are examined using longitudinal data.

### 2.1. Hypotheses

With respect to the hypotheses for the first aim, recent research has shown that NGRNs’ satisfaction with their work environment tends to remain stable during the first year, whereas their turnover intention tends to increase around 6 months after employment (or, conversely, their intention to stay tends to decrease). However, as NGRNs’ work‐related support and job responsibilities gradually shift to typical clinical levels over the course of the training program, they may perceive a relative decrease in job resources, which may lead to a decline in satisfaction with the work environment. Therefore, the first and second hypotheses are proposed as follows:

 
**Hypothesis 1:** NGRNs’ satisfaction with the work environment tends to be stable or decreases across the three stages of the two‐year on‐the‐job training program.
 
**Hypothesis 2:** NGRNs’ turnover intention tends to increase (or, conversely, their intention to stay tends to decrease) across the three stages of the two‐year on‐the‐job training program.

With respect to NGRNs’ individual resilience, according to the JD–R and COR models, this characteristic may be fostered by job resources, such as satisfaction with the work environment, as well as by nurses’ skills and experience developed through the on‐the‐job training program. In addition, higher individual resilience is associated with lower turnover intention or greater intention to stay. However, recent research has shown that satisfaction with the work environment tends to remain relatively stable over time during the on‐the‐job training program period, whereas turnover intention tends to increase. Therefore, even when changes in job resources do not substantially affect NGRNs’ satisfaction with the work environment, they may still be insufficient to promote—or may even undermine—the development of individual resilience, thereby contributing to increased turnover intention. On the basis of this reasoning, the third and fourth hypotheses are proposed as follows:

 
**Hypothesis 3:** NGRNs’ individual resilience tends to remain stable or **decrease** across the three stages of the two‐year on‐the‐job training program.
 
**Hypothesis 4:** During NGRNs’ two‐year on‐the‐job training program, satisfaction with the work environment will positively predict individual resilience. Furthermore, both variables will positively predict intention to stay (or negatively predict turnover intention).

With respect to the hypotheses for the second aim, previous studies and the JD–R model suggest that individual resilience may mediate the relationship between satisfaction with the work environment and turnover intention (or the intention to stay) and that this mediating relationship may also be valid across the stages of the two‐year on‐the‐job training program. Therefore, the fifth hypothesis is proposed as follows:

 
**Hypothesis 5:** The mediating effect of individual resilience among NGRNs remains valid across the two‐year on‐the‐job training program.

## 3. Methods

### 3.1. Design

This study was conducted between December 1, 2020, and June 28, 2024, and employed a longitudinal design with convenience sampling to survey NGRNs using questionnaires at three time points, corresponding to the completion of each stage of the two‐year on‐the‐job training program (3 months, 1 year, and 2 years after onboarding).

The first survey served as the baseline and was conducted after the NGRNs completed the first stage of the training program (the basic curriculum stage). At this stage, the NGRNs had acquired a basic understanding of their work roles and had begun preliminary clinical practice under close supervision and guidance, which allowed for an initial evaluation of working conditions. Relevant resources were expected to be at an early stage of development, which was consistent with the aims of our study. This time point has commonly been used as a baseline survey time point or as an inclusion criterion in studies conducted in Taiwan that examine the impact of nursing work conditions on nurses on the basis of similar methodological considerations [42, 46, 48].

With respect to the subsequent survey time points, according to the JD–R and COR models, resources require time to develop [13, 16, 18], and different resources may develop at different rates. In addition, Stages 2 and 3 of the on‐the‐job training program involve learning through step‐by‐step practice; therefore, the effects of the training intervention are expected to emerge in the later phases of the program. Because our study focused on the effects of the on‐the‐job training program across different stages rather than on short‐term fluctuations, we selected the end of each training stage as the follow‐up measurement time points. At these time points, NGRNs were expected to have adapted to the working conditions and the training program for several months, and the characteristics of interest were therefore assumed to be relatively more stable.

### 3.2. Participants

All participants in this study were registered nurses working at a medical center in northern Taiwan who had graduated less than 1 year prior with no work experience or who had graduated less than 1 year prior and had brief nursing experience in another medical facility. Both groups were regarded as NGRNs, were required to participate in the two‐year on‐the‐job training program, and had just completed the first 3 months of the first stage of the two‐year on‐the‐job training program.

### 3.3. Recruitment Process

First, the researchers contacted the hospital’s nursing department to obtain permission to recruit NGRNs. Second, after obtaining consent from the department, the researchers requested a monthly list of NGRNs who had completed their initial staff training and their contact information (the first stage of the two‐year on‐the‐job training), and then recruited them into this study. This recruitment process lasted for 2 years (from June 1, 2020, to July 1, 2022).

During recruitment, the researchers contacted potential participants on the basis of the monthly list and visited them in their departments at convenient times to introduce the study and invite them to participate. NGRNs who agreed to join the study received a baseline questionnaire and were followed up twice: 1 year after employment when they had completed the second stage of training and 2 years after employment when they had completed the final stage of training.

The questionnaire received by the NGRNs for each wave was placed in an opaque, sealable envelope along with a flyer providing detailed study information and a small gift worth approximately 100 NTD to thank them for their participation and to encourage continued involvement. Participants were asked to complete the questionnaire within 1 week, seal it in the envelope after completion, and either place it in a designated collection box in their department or return it directly to the researcher. The researchers collected the completed questionnaires 1 week after they were distributed. If a questionnaire was not received, the researchers reminded the participant to submit it. If the participant still did not submit the questionnaire, the researchers continued to send weekly reminders and check whether it had been submitted. If the questionnaire was not received after 1 month, the participant was considered to have withdrawn from the study.

### 3.4. Instruments Used in This Research, Including Their Validity and Reliability

In terms of content, the questionnaire used in this study consisted of 4 parts: the participants’ demographic information, resilience, satisfaction with the work environment, and intention to stay.

#### 3.4.1. Participants’ Background Information

We collected background information that may affect nurses’ resilience and work environment, including their age, marital status, level of education, work department, clinical level, and exercise habits. The clinical ladder reflects the level of nursing experience within the on‐the‐job training program. The N level refers to NGRNs, whereas the N1 level refers to nurses with more than 1 year of clinical experience who have passed the general nursing knowledge examination. In our study, we included a small number of nurses who had brief prior nursing experience in another medical facility and who obtained N1‐level qualifications within the first 3 months of employment. These nurses were still classified as NGRNs and were reenrolled in the on‐the‐job training program.

#### 3.4.2. Individual Resilience

The 25‐item Connor–Davidson Resilience Scale (CD‐RISC), which was developed by Connor and Davidson [49], was used in this study. Initially, this scale was designed to measure the psychological resilience exhibited by individuals with posttraumatic stress disorder or anxiety; however, it can also be used to assess resilience in healthy individuals [50]. The measure is scored on a 5‐point Likert scale on which individuals indicate the degree of agreement between their own experiences and the items, using scores ranging from 0 (*not true at all*) to 4 (*true nearly all the time*). The total score ranges from 0 to 100. Higher scores indicate higher levels of resilience. This scale has been translated into traditional Chinese and has previously been used in surveys of nurses [51–53].

With respect to the reliability and validity of this scale, no validity studies have been reported among nurses in Taiwan. However, Hsu et al. [51] reported good reliability with a Cronbach’s alpha coefficient of 0.95 on the basis of data collected from 130 nurse practitioners at a medical center in southern Taiwan. In our study, univariate construct validity was assessed using the comparative fit index (CFI). The CFI values across all three survey waves ranged from 0.931 to 0.942, indicating that the construct validity of this scale was acceptable. Cronbach’s alpha coefficient for all three waves was 0.95, demonstrating high internal consistency for this scale. This measure was therefore applicable to our participants.

#### 3.4.3. Satisfaction With the Work Environment

Satisfaction with the work environment was measured via the Taiwanese version of the Revised Individual Workload Perception Scale (T‐IWPS‐R), which was adapted from the Individual Workload Perception Scale [19, 54, 55] and translated into traditional Chinese for Taiwan by Lin et al. [55]. This scale includes five subgroups: manager support, peer support, unit support, workload, and intention to stay. The original scale contains a total of 29 items, whereas the Taiwanese version includes 24 items [55]. This measure is scored on a 5‐point Likert scale on which individuals indicate their level of agreement with each item on the basis of their own experiences; scores range from 1 (*strongly disagree*) to 5 (*strongly agree*). The average score for each item within a subgroup is typically used to represent the result for the subgroup in question. Higher scores indicate higher levels of satisfaction with the work environment.

With respect to the reliability and validity of this scale, Lin et al. [55] reported that the content validity index (CVI) was 0.93 as assessed by five experts, including two nursing faculty members, one pharmacist, one nurse administrator, and one clinical researcher. The internal consistency of the scale, as measured by Cronbach’s alpha, was 0.88 for the total scale, with coefficients ranging from 0.61 to 0.85 for the subscales on the basis of data collected from 344 nurses who provided direct patient care at a regional teaching hospital in southern Taiwan [55]. In our study, Cronbach’s alpha coefficients for the whole scale ranged from 0.92 to 0.93 across all three waves of the survey. With respect to the subscales, Cronbach’s alpha coefficients ranged from 0.61 to 0.87 across the three waves. Therefore, this scale was applicable to our participants.

Since this study explored the relationships among satisfaction with the work environment, individual resilience, and turnover intention, the subscale that measured intention to stay was separated from the construct of satisfaction with the work environment and treated as a reverse indicator of turnover intention. The score for satisfaction with the work environment was calculated as the average of the remaining subscale scores. With respect to the impact on the validity of the Satisfaction with the Work Environment Scale, we examined criterion validity by analyzing the correlation coefficients between the scales with and without the intention‐to‐stay items. The results showed high correlations across the three survey waves (Wave 1: *r* = 0.95, *p* < 0.001; Wave 2: *r* = 0.97, *p* < 0.001; Wave 3: *r* = 0.96, *p* < 0.001), indicating that the impact on validity was minimal.

#### 3.4.4. Turnover Intention/Intention to Stay

In this study, we used the intention‐to‐stay subscale of the measure of satisfaction with the work environment as a reverse indicator of turnover intention. Higher scores on the intention‐to‐stay subscale indicate stronger intention to remain in one’s current job and thus weaker turnover intentions. Cronbach’s alpha coefficients for this scale ranged from 0.74 to 0.84 across the three waves, indicating that the scale exhibited good internal consistency and was suitable for our study.

### 3.5. Inclusion and/or Exclusion Criteria

#### 3.5.1. Inclusion Criteria

The participants in this study were registered nurses who had just completed the first stage of the two‐year on‐the‐job training program.

#### 3.5.2. Exclusion Criteria

Participants whose questionnaires contained at least one scale for which more than half of the items remained unanswered were excluded from this research. In addition, participants who requested to withdraw from the study, did not participate fully in all three waves of the survey, or were reassigned to non‐nursing departments during the study period were excluded from this research. However, the first‐wave data provided by these participants were used to analyze the differences between this group and those who participated fully in this research (if they completed the first‐wave surveys).

### 3.6. Efforts to Address Missing Values

To address missing values in the scales, missing values were filled with the average score of the corresponding item depending on the wave of the survey. With respect to missing values in the background section, questionnaires that exhibited missing values were excluded when the analysis focused on those items.

### 3.7. Data Analysis

The data referenced in this study were cleaned and adjusted with the assistance of Microsoft Excel and analyzed using R software (Version 4.4.3). The methods used for data analysis are described below.

Descriptive statistics were used to present the participants’ background information and to provide an initial examination of the trends that characterized the changes observed in all scale scores.

To analyze the differences between nurses who participated fully in this research and those who withdrew, the chi‐square test was used to analyze the categorical background variables for these two groups. Continuous variables (e.g., age and scale scores) were analyzed on the basis of an independent *t*‐test with the goal of examining the differences between these two groups.

To address the first aim and Hypotheses 1–3 of this study, two analytical methods were employed. First, a one‐way repeated‐measures analysis of variance (ANOVA) was conducted to initially examine whether the scale scores differed significantly across the survey waves. In this approach, if the scale scores did not satisfy the sphericity assumption, the Huynh–Feldt correction was applied to adjust the *p* value. Additionally, post hoc analyses were performed using the Bonferroni method to adjust the *p* values for multiple paired *t*‐tests. Second, linear mixed models (LMMs) were employed to examine whether the scale scores showed significantly increasing or decreasing trends across the three survey waves. In this approach, the survey waves were treated as a continuous variable to evaluate the significance of the temporal trends in the scale scores. In addition, random slopes for waves were included in the LMMs to account for individual variability in these longitudinal changes.

With respect to the analysis of the relationships between background variables and scale scores, an LMM was employed. In this approach, each background variable was included separately in the model, and the effects of different waves of the survey were adjusted. For variables that were associated with more than two categories, post hoc pairwise comparisons of estimated marginal means were conducted according to the procedures suggested by Searle et al. [56] and Hothorn et al. [57], and the resulting *p* values were adjusted via the Tukey method. The background variables that had significant effects on the outcomes were included in the models to adjust for potential confounding effects.

To address the second aim and Hypotheses 4 and 5, we examined the relationships among the work environment, individual resilience, and intention to stay as well as the mediating effects among these characteristics. These effects were analyzed via the method proposed by Baron and Kenny [58]. First, we tested whether both the predictor variable and the mediator variable individually predicted the outcome variable. Second, we investigated whether the predictor variable predicted the mediator variable. Finally, we entered both the predictor and the mediator into the same model to predict the outcome variable and assessed how the predictive power of each variable changed. In addition, the direct, indirect, and total effects were estimated via the mediation package for R [59], which implements the quasi‐Bayesian Monte Carlo simulation approach developed by Imai et al. [60]. With respect to hierarchical data such as our repeated‐measures dataset, the package reports cluster averages of the average causal mediating effects and average direct effects in line with the suggestions of Imai and Yamamoto [61]. We used 5000 simulation draws, following the recommendations of Preacher and Hayes [62].

### 3.8. Sample Size

Since this study featured a longitudinal design and employed LMMs to adjust for time effects and analyze the mediating effects among the outcomes, we referred to Maas and Hox [63] and Meteyard and Davies [64], who reported that more than 50 clusters are needed to ensure adequate statistical power and minimize estimation bias. Additionally, McNeish and Stapleton (2016) recommended that more than 100 clusters should be included to ensure a more stable estimation of fixed effects. Therefore, our study included more than 100 clusters to ensure sufficient power and stability.

### 3.9. Declaration on the Use of Artificial Intelligence (AI)–Generated Content

The content of this article was not generated by AI tools. AI tools were used in this article only to assist the authors in correcting English grammar to ensure that the sentences accurately convey the intended meaning.

### 3.10. Ethical Considerations

This study was approved by the Institutional Review Board of Taipei Tzu Chi Hospital (case number: iIRB No. 09‐XD‐091) on October 19, 2020. All the participants were fully informed of the purpose of this study by the researcher and agreed to participate in this research. Participation in this study was entirely voluntary, and participants could withdraw from this research at any time without providing a reason.

In terms of data storage, the collected paper questionnaires were stored in a locked cabinet. After the data from the questionnaires were entered into the principal investigator’s encrypted computer in the office, the paper questionnaires were destroyed. In addition, the personal information section of the questionnaire was coded upon entry into the computer to ensure that the files from the three surveys could be linked while maintaining the security of the participants’ data.

## 4. Results

A total of 200 NGRNs were invited to participate in this study, of whom 195 agreed to participate in this research. In the second wave, 166 nurses remained, and 144 fully completed all three waves of the survey, thus satisfying the sample size requirement of 100 participants.

The most common reason participants withdrew from this study was resignation: 24 participants (82.8%) resigned after the 1st wave of the survey, and 16 participants (72.7%) resigned after the 2nd wave of the survey. Other reasons participants withdrew from this study included reassignment to non‐nursing departments and requests to leave the study. Since the participants could withdraw from this research at any time without providing a reason, the precise reasons for participants’ requests to leave the study are unknown.

### 4.1. Background Information Concerning the Participants

The results are presented in Table 1. The participants’ average age was 21.72 ± 1.33 years, and they had approximately half a year of nursing experience (0.56 ± 0.54 years). Most of the participants were female (86.8%), were not responsible for caring for older adults at home (72.9%), had graduated from junior college (66.7%), worked in intensive and critical care units (35.4%), were at the N level (98.6%) of the clinical ladder, and reported no exercise habits (72.2%).

**TABLE 1:** Participants’ background and comparison between those who completed and those who withdrew from the study (*N* = 195).

Variables	Full participated (*n* = 144)	Withdrew (*n* = 51)	*p*
	*N* (%)/mean ± S.D.	*N* (%)/mean ± S.D.	
Age	21.72 ± 1.33	22.84 ± 2.55	0.003^a^ ^∗^
Sex			0.584^b^
Female	125 (86.8%)	42 (82.4%)	
Male	19 (13.2%)	9 (17.6%)	
Need caring older at home			0.556^b^
No	105 (72.9%)	40 (78.4%)	
Yes	39 (27.1%)	11 (21.6%)	
Education			0.513^b^
Junior college	96 (66.7%)	20 (39.2%)	
University	48 (33.3%)	31 (60.8%)	
Nursing experience (years)	0.56 ± 0.54	0.37 ± 0.27	0.100^a^
Work department			0.179^b^
Medical wards	35 (24.3%)	18 (35.3%)	
Surgical wards	30 (20.8%)	7 (13.7%)	
Psychiatric ward	2 (1.4%)	3 (5.9%)	
Intensive and critical care units	51 (35.4%)	12 (23.5%)	
Pediatric wards	4 (2.8%)	1 (2.0%)	
Operating rooms	20 (13.9%)	10 (19.6%)	
Outpatient departments	2 (1.4%)	0 (0.0%)	
Exercise habits			0.894^b^
No	104 (72.2%)	38 (74.5%)	
Yes	40 (27.8%)	13 (25.5%)	
Resilience	63.25 ± 14.12	57.26 ± 15.08	0.012^a^ ^∗^
Work environment	4.14 ± 0.48	3.94 ± 0.38	0.006^∗^
Manager support	4.15 ± 0.52	4.13 ± 0.43	0.816^a^
Peer support	4.32 ± 0.50	4.16 ± 4.32	0.052^a^
Unit support	4.27 ± 0.56	4.11 ± 0.44	0.038^a^ ^∗^
Workload	3.83 ± 0.70	3.77 ± 0.74	0.568^a^
Intent to stay	3.39 ± 0.70	3.15 ± 0.62	0.035^a^ ^∗^

*Note:* These results present a comparison of background variables between retained and withdrawn participants, based on data from the first survey.

^a^Analyses were conducted using independent *t*‐tests or one‐way ANOVA.

^b^Analyses were conducted using chi‐square.

^∗^ *p* < 0.05.

^∗∗^ *p* < 0.001.

In terms of the participants’ average scores on the scales used in this research, the average resilience score was 63.25 ± 14.12, the average work environment score was 4.17 ± 0.47, and the average intention to stay score was 3.39 ± 0.70.

### 4.2. Results of the Analysis of the Differences Between Full Participants and Those Who Withdrew From This Research

The results are presented in Table 1. The proportional distributions and means of most background variables did not differ between full participants and those who withdrew from this research, except that the latter were slightly older than those who participated fully in this research (fully participated: 21.72 ± 1.33; withdrawn: 22.84 ± 2.55; *p* < 0.05).

In our results, participants who withdrew from this research exhibited lower levels of resilience (fully participated: 63.25 ± 14.12; withdrawn: 57.26 ± 15.08; *p* < 0.05), satisfaction with their work environment (fully participated: 4.14 ± 0.48; withdrawn: 3.94 ± 0.38; *p* < 0.05), and intention to stay in their job (fully participated: 3.39 ± 0.70; withdrawn: 3.15 ± 0.62; *p* < 0.05).

### 4.3. Validating Hypotheses

#### 4.3.1. Differences in Scale Scores Across the Waves of the Survey

The first aim and Hypotheses 1–3 concerned changes in individual resilience, satisfaction with the work environment, and intention to stay across the three survey waves in NGRNs. The results are presented in Table 2.

**TABLE 2:** Results of different analyses across three survey waves in satisfaction with the work environment, individual resilience, and intention to stay (*n* = 144).

**Results of repeated measures ANOVA**				
**Variables**	**1**st **wave**	**2**nd **wave**	**3**rd **wave**	**p**
	**Mean ± S.D.**	**Mean ± S.D.**	**Mean ± S.D.**	

Resilience	63.25 ± 14.12	63.85 ± 13.82	65.84 ± 14.58	0.074
Work environment	4.14 ± 0.48	3.98 ± 0.48	3.88 ± 0.48	< 0.001^a^ ^∗∗^
Manager support	4.15 ± 0.52	4.05 ± 0.48	3.94 ± 0.50	< 0.001^a^ ^∗∗^
Peer support	4.32 ± 0.50	4.21 ± 0.72	4.11 ± 0.49	0.002^b^ ^∗^
Unit support	4.27 ± 0.56	4.01 ± 0.55	4.00 ± 0.63	< 0.001^c^ ^∗∗^
Workload	3.83 ± 0.70	3.66 ± 0.72	3.48 ± 0.78	< 0.001^a^ ^∗∗^
Intent to stay	3.39 ± 0.70	3.15 ± 0.65	2.95 ± 0.76	< 0.001^a^ ^∗∗^

**Results of linear mixed model**				
**Variables**	***β***	**S.E.**	***t* value**	**p**

Resilience	1.30	0.64	2.04	0.043^∗^
Work environment	−0.12	0.02	−6.06	< 0.001^∗∗^
Manager support	−0.16	0.02	−4.64	< 0.001^∗∗^
Peer support	−0.11	0.03	−3.76	< 0.001^∗∗^
Unit support	−0.14	0.03	−4.99	< 0.001^∗∗^
Workload	−0.17	0.04	−4.85	< 0.001^∗∗^
Intent to stay	−0.22	0.04	−6.01	< 0.001^∗∗^

*Note:* The results of the linear mixed model treated the wave variable as a continuous variable. Work environment: The average score of satisfaction with the work environment scale, excluding the “intention to stay” subscale.

^a^Scores across these three waves were all significantly different.

^b^Scores in the 1st wave were significantly higher than those in the 3rd wave.

^c^Scores in the 1st wave were significantly higher than those in the other waves.

^∗^ *p* < 0.05.

^∗∗^ *p* < 0.001.

With respect to the results regarding individual resilience, the mean scores observed across the survey waves exhibited an upward trend; therefore, we used the LMM results as our primary results. In the analysis, the repeated‐measures ANOVA indicated only marginal significance (1st wave = 63.25 ± 14.12, 2nd wave = 63.85 ± 13.82, 3rd wave = 65.84 ± 14.58, *p* = 0.074), whereas the LMM results indicated a statistically significant upward trend (*β* = 1.30, S.E. = 0.64, *p* < 0.05). These results suggested that the differences in individual resilience across the waves were not substantial, but the scores nevertheless showed a significantly increasing trend over time, which differed from our hypothesis.

With respect to the results concerning the work environment, the mean scores observed across the survey waves exhibited a downward trend. In the analysis, both the repeated‐measures ANOVA and the LMM indicated a significant decreasing trend in the corresponding scores across the survey waves (repeated‐measures ANOVA: 1st wave = 4.14 ± 0.48, 2nd wave = 3.98 ± 0.48, 3rd wave = 3.88 ± 0.48, *p* < 0.001; LMM: *β* = −0.12, S.E. = 0.02, *p* < 0.001), including the subscale scores. These results indicated increasing dissatisfaction with the work environment among the participants, which differed from our hypothesis.

With respect to the results regarding the intention to stay, the mean scores observed across the survey waves exhibited a downward trend; therefore, we used the LMM results as our primary results. In addition, the results of both the repeated‐measures ANOVA and the LMM revealed that this trend was significant (repeated‐measures ANOVA: 1st wave = 3.39 ± 0.70, 2nd wave = 3.15 ± 0.65, 3rd wave = 2.95 ± 0.76, *p* < 0.001; LMM: *β* = −0.22, S.E. = 0.04, *p* < 0.001). These results indicated that the participants’ intention to stay decreased over time, which supports our hypothesis.

#### 4.3.2. Validation of Hypothesis 4 and Mediation Analysis

The second aim, along with Hypotheses 4 and 5, proposed that during the on‐the‐job training period, NGRNs’ satisfaction with their work environment would positively predict individual resilience, and both variables would positively predict intention to stay (or negatively predict turnover intention). In addition, individual resilience would mediate the effect of satisfaction with the work environment on turnover intention (or intention to stay). In this approach, we also adjusted for the background variables that significantly affected the scale scores. For individual resilience (need vs. does not need: *β* = −3.27, S.E. = 1.48, *p* < 0.05) and intention to stay (need vs. does not need: *β* = −0.17, S.E. = 0.08, *p* < 0.05), we adjusted for the variable “responsible for caring for elderly family members.” For satisfaction with the work environment, we adjusted for age (each additional year: *β* = −0.05, S.E. = 0.02, *p* < 0.05) and education (university vs. junior college: *β* = −0.19, S.E. = 0.06, *p* < 0.001). The results are presented in Table 3.

**TABLE 3:** Mediation analysis of resilience, work environment, and intention to stay (*n* = 144).

Variables	Outcome variables							
	Resilience		Intent to stay					
	Model 1		Model 2		Model 3		Model 4	
	*β* (S. E.)	*p*	*β* (S. E.)	*p*	*β* (S. E.)	*p*	*β* (S. E.)	*p*
WE	11.42 (1.24)	< 0.001^∗∗^	0.59 (0.07)	< 0.001^∗∗^	—	—	0.51 (0.07)	< 0.001^∗∗^
Resilience	—	—	—	—	0.01 (0.002)	< 0.001^∗∗^	0.006 (0.002)	0.008^∗^
wave	2.77 (0.60)	< 0.001^∗∗^	−0.14 (0.04)	< 0.001^∗∗^	−0.24 (0.04)	< 0.001^∗∗^	−0.16 (0.04)	< 0.001^∗∗^

*Mediation effect analysis*								
Indirect effect: 0.08, 95%CI = 0.02 ∼ 0.14, *p* = 0.007^∗^								
Direct effect: 0.52, 95%CI = 0.37 ∼ 0.65, *p* < 0.001^∗∗^								
Total effect: 0.60, 95%CI = 0.46 ∼ 0.73, *p* < 0.001^∗∗^								
Proportion of mediating effects: 0.13, 95%CI = 0.03 ∼ 0.24, *p* = 0.007^∗^								

*Note:* In all of the models, we adjusted for the “need to care for an older adult at home” variable, which was found to affect individual resilience and intention to stay, based on the results of the analysis of relationships between background variables and scale scores. WE: satisfaction with the work environment; resilience: individual resilience.

Abbreviation: CI, confidence interval.

^∗^ *p* < 0.05.

^∗∗^ *p* < 0.001.

With respect to Hypothesis 4, Models 1–3 revealed that the participants’ satisfaction with their work environment significantly predicted individual resilience even after adjusting for background variables (*β* = 11.42, S.E. = 1.24, *p* < 0.001), indicating that a one‐point increase in satisfaction with the work environment was associated with an 11.42‐point increase in individual resilience. In addition, both individual resilience (*β* = 0.01, S.E. = 0.002, *p* < 0.001) and satisfaction with the work environment (*β* = 0.59, S.E. = 0.07, *p* < 0.001) significantly predicted participants’ intention to stay. Specifically, a one‐point increase in individual resilience was associated with a 0.01‐point increase in intention to stay, whereas a one‐point increase in satisfaction with the work environment was associated with a 0.59‐point increase in intention to stay. It is worth noting that the scale ranges of individual resilience (0–100) and intention to stay (1–5) are different. After transforming both scales to a common range (0–100), a one‐point increase in individual resilience was associated with a 0.25‐point increase in intention to stay, which is smaller than the 0.59‐point increase associated with satisfaction with the work environment. These results support Hypothesis 4.

With respect to Hypothesis 5, we analyzed the mediating effect. First, our findings indicated that participants’ satisfaction with the work environment and individual resilience (*β* = 0.01, S.E. = 0.002, *p* < 0.001) both independently and significantly predicted their intention to stay. Second, satisfaction with the work environment also significantly predicted individual resilience (*β* = 11.42, S.E. = 1.24, *p* < 0.001). Finally, when both the work environment and individual resilience were entered into the same predictive model, each factor remained a significant predictor of the participants’ intention to stay (work environment [*β* = 0.51, S.E. = 0.07, *p* < 0.001] and individual resilience [*β* = 0.006, S.E. = 0.002, *p* < 0.05]), although their parameter estimates decreased. According to the criteria proposed by Baron and Kenny [58], these results suggest that individual resilience partially mediates the effect of the work environment on the intention to stay.

Furthermore, we analyzed the indirect, direct, and total effects in the mediation model. Our results revealed that the average causal mediating effect (indirect effect) was 0.08 (95% CI: 0.02∼0.14, *p* < 0.05), the direct effect was 0.52 (95% CI: 0.37∼0.65, *p* < 0.001), and the total effect was 0.60 (95% CI: 0.46∼0.73, *p* < 0.001), thus demonstrating that individual resilience significantly mediated the relationship between the work environment and the intention to stay. Approximately 13% (95% CI: 0.03∼0.24) of the effect of the work environment on the intention to stay was mediated by individual resilience (*p* < 0.05). These results support Hypothesis 5.

## 5. Discussion

The aims of this study were to investigate changes in NGRNs’ satisfaction with the work environment, individual resilience, and intention to stay across the two‐year on‐the‐job training program implemented by the Taiwanese government and to examine the relationships among these three characteristics, including the proposed mediation effects. Overall, these aims were largely achieved: Our results supported Hypotheses 1, 2, 4, and 5 but did not support Hypothesis 3.

**Hypothesis 1** proposed that NGRNs’ satisfaction with their work environment would tend to remain stable or decrease across the stages of the training program. Our results showed that this characteristic exhibited a significant decreasing trend across the three survey waves. Since satisfaction with the work environment reflects how well the work environment supports nurses in performing their nursing practice [19–21], as the on‐the‐job training program progressed, NGRNs gradually began to perform their duties more independently. During this process, support from preceptors and leaders gradually decreased to typical levels, whereas work responsibilities and workload increased, particularly in the third stage, which involved professional nursing practice and more complex tasks. Consequently, these changes may have led NGRNs to perceive decreased support from their work environment and increased workload, resulting in a decline in satisfaction with the work environment, which may help to explain our findings.

However, our results differed from those of Liu et al. [46], who reported that satisfaction with the work environment remained stable during the first year. In their study, approximately 30% of participants transferred to other hospitals or medical facilities during the survey period. These participants may have experienced longer periods of lower workload or earlier stages of the training program, potentially leading to an underestimation of changes associated with the program. Notably, Liu et al. [46] also reported a decreasing trend in satisfaction with the work environment, although this trend did not reach statistical significance. Taken together, these factors may help to explain the discrepancy between their findings and ours.

**Hypothesis 2** proposed that NGRNs’ turnover intention would tend to increase (or, conversely, that their intention to stay would tend to decrease) across the stages of the training program. Our results supported this hypothesis. According to the JD–R model, when job resources and personal resources are insufficient to meet job demands, positive job outcomes tend to decline [13], including the intention to stay. In addition, the COR model suggests that when individuals perceive their expected resources to be threatened, they are motivated to take action to mitigate perceived resource loss [18]. Therefore, as the on‐the‐job training program progresses and NGRNs experience reduced job resources alongside increased job demands (reflected in decreased support and increased workload), they may develop intentions to reduce or mitigate perceived resource loss, which may manifest as a decline in the intention to stay.

Our results also align with those of Su et al. [45], who reported that newly employed nurses from six hospitals exhibited a significant increase in turnover intention 6 months after employment. Similarly, Chen et al. [44] reported that NGRNs had a higher turnover rate during the second stage of the on‐the‐job training program, which may be attributable to the requirement to work more independently, reduced perceived support for job performance, and increased workload, ultimately leading to turnover as a strategy to avoid perceived threats to core resources. Although our study did not observe an increase in actual turnover rates among participants, we identified a progressive effect of the on‐the‐job training program and extended the findings of Su et al. [45] to the second year after employment because we observed that NGRNs’ intention to stay (the converse of turnover intention) tended to decrease over time.

Notably, our study period overlapped with the COVID‐19 pandemic. During this time, Taiwan experienced two major outbreaks [65, 66]. Nagel et al. [67] reported that medical staff may have experienced a lack of medical resources, fear of infection, and other adverse working conditions during the COVID‐19 pandemic. In addition, Liao et al. [68] reported that during the pandemic, hospital response mechanisms concentrated resources on pandemic prevention, which negatively affected nurses’ satisfaction with the work environment in units that were not dedicated to COVID‐19. Although most NGRNs were not directly involved in frontline COVID‐19 care and the surveys in this study were conducted several months after the outbreaks, pandemic‐related working conditions and hospital response mechanisms may have led NGRNs to perceive decreased job resources and increased job demands, thereby affecting their satisfaction with the work environment and their intention to stay. These factors may have contributed to an overestimation of the observed trends during the on‐the‐job training program. Although this may not affect the main theoretical interpretation, it may have amplified the observed trends and, therefore, should be considered a limitation of this study.

**Hypotheses 3 and 4** proposed that NGRNs’ individual resilience would tend to remain stable or decrease across the three stages of the two‐year on‐the‐job training program and that NGRNs’ satisfaction with the work environment would positively predict individual resilience. Furthermore, both variables would positively predict intention to stay across the on‐the‐job training program. Interestingly, our results did not support Hypothesis 3 but did support Hypothesis 4. Specifically, although the three variables positively predicted one another among our participants, individual resilience increased over time even as satisfaction with the work environment and the intention to stay decreased.

According to the JD–R and COR theories, individual resilience can be conceptualized as a personal resource or as an indicator of personal resources, and the work environment represents an important antecedent that can foster resilience and influence job outcomes, such as turnover intention or the intention to stay [11, 13, 18, 26, 29]. Given that our results revealed a decline in satisfaction with the work environment and the intention to stay across the on‐the‐job training program, the development of individual resilience may have been constrained by a decrease in job resources. Under such conditions, the buffering effect of individual resilience on the intention to stay may have been limited. Notably, contrary to our expectations, individual resilience increased over time.

A possible explanation is that personal resources reflect positive self‐evaluations of individuals’ ability to control and successfully influence their environment [13]. In addition to satisfaction with the work environment, nursing practice experience and structured training courses provided during the on‐the‐job training program may function as job resources that are not fully captured by the work environment measure. These resources may enhance NGRNs’ work‐related competencies and thereby foster the development of personal resources, such as individual resilience, which can be conceptualized as a personal resource or an indicator of personal resources [13, 29]. As a result, even though satisfaction with the work environment decreased over time, individual resilience continued to increase. This interpretation may also help to explain why individual resilience exhibited a significant overall increasing trend across the on‐the‐job training program, whereas the mean differences between adjacent survey waves were only marginally significant, suggesting that diminishing job resources may have attenuated the rate of resilience development. These results are consistent with those of previous studies. Some studies have shown that nurses’ individual resilience is associated with turnover intention [30, 33, 36, 40, 42, 47], whereas others have reported that satisfaction with the work environment is associated with turnover intention [30, 33, 35, 36, 69]. In addition, several studies have reported that these three variables are interrelated [11, 21, 26, 36]. Furthermore, prior research has shown that individual resilience among nurses is associated with nursing experience [11, 26, 39] and with work experience in the same workplace [11, 26, 70, 71]. Hartmann et al. [11] reported that individuals who possess job‐related expertise or are able to manage work demands effectively tend to exhibit higher levels of individual resilience.

It is worth noting that, although participants’ individual resilience increased across the on‐the‐job training program, their intention to stay decreased. In addition, our LMM results indicated that the effect of a one‐point increase in individual resilience on intention to stay was approximately half of that of a one‐point increase in satisfaction with the work environment. Given that nursing is widely recognized as a high‐stress profession [5, 6] and that nursing shortages have become a pressing global issue in recent years [2, 3], any intervention that can consistently improve retention or reduce turnover intention should not be overlooked, even if its effect is modest. Therefore, although the benefit of enhancing NGRNs’ individual resilience is smaller than that of improving their work environment, it remains meaningful. However, these findings also suggest that enhancing individual resilience alone may be insufficient to sustain intention to stay. As suggested by previous studies [24, 72], resilience can reduce negative job outcomes among nurses; however, problems in the nursing work environment should not be ignored. Taken together, these findings help explain why individual resilience positively predicts intention to stay, whereas their longitudinal trends move in opposite directions. They further indicate that effectively improving NGRNs’ intention to stay requires both enhancing the work environment and strengthening individual resilience.

**Hypothesis 5** proposed that NGRNs’ individual resilience would mediate the effect of satisfaction with the work environment on turnover intention (or intention to stay). According to the JD–R model, job resources promote the development of personal resources, and personal resources play a mediating role in influencing job outcomes [13, 15, 16]. Because satisfaction with the work environment represents job resources, individual resilience is a personal resource, and the intention to stay is a job outcome, individual resilience may mediate the effect of satisfaction with the work environment on the intention to stay. Our results supported this hypothesis and showed that approximately 13% of the effect of satisfaction with the work environment on the intention to stay was mediated by individual resilience across the survey waves. According to COR theory, when individuals perceive a threat to their resources, they tend to take action to mitigate this threat [18], which is consistent with our findings. Thus, although NGRNs’ individual resilience tended to increase and continued to provide a buffering effect, the threats to core resources caused by job resources and job demands returning to typical levels as the on‐the‐job training program progressed may have been strong enough to reduce their intention to stay as a strategy to mitigate perceived resource loss. Our results are consistent with those of other studies that have shown that satisfaction with the work environment is an antecedent of individual resilience among nurses and that individual resilience is a protective factor against turnover [11, 26]. Yu and Lee [36] surveyed 371 NGRNs in Korea and reported that their individual resilience mediated the effect of satisfaction with the work environment on turnover intention. A study [69] with results that differed from ours surveyed NGRNs and reported that only satisfaction with the work environment affected their intention to stay, whereas individual resilience was correlated only with the intention to stay. However, their results were based on a multiple regression model that included all predictors (e.g., satisfaction with the work environment, individual resilience, and self‐efficacy) simultaneously to predict the intention to stay. Given this modeling approach, mediating mechanisms or shared explanatory variance among these variables—particularly between self‐efficacy, which is a personal resource, and individual resilience, which can be regarded as an indicator of personal resources—may have contributed to the differences between their results and our findings.

### 5.1. Differences Between Participants Who Participated Fully and Participants Who Withdrew From This Research

Our results revealed that participants who withdrew from our study reported lower levels of satisfaction with the work environment, resilience, and intention to stay than did participants who participated fully in our study. These two groups of participants were similar in terms of background variables; the only difference was that participants who withdrew were older than those who completed all waves of the survey. We observed that most of the reasons for participants’ withdrawal from our study were related to leaving their job, which is consistent with the findings reported by previous studies [11, 26, 41, 73, 74]. Bae [73] summarized reports concerning the factors that affect NGRNs, and Ulrich et al. [74] summarized reports concerning the factors that affect nurses’ turnover rates and revealed that work environment–related factors and the intention to stay can predict turnover rates among NGRNs and other nurses. Lee and De Gagne [41] followed up with NGRNs for 1 year and reported that individual resilience significantly predicted turnover rates among NGRNs. On the basis of our results and those of previous studies, monitoring these characteristics is important to prevent turnover among NGRNs.

### 5.2. Implications

Although our results indicate that the two‐year on‐the‐job training program can effectively promote NGRNs’ individual resilience to buffer against subsequent job demands in their nursing practice, the progression of the training program is accompanied by a reduction in job resources and an increase in job demands. Previous studies suggest that a workplace culture characterized by effective communication and collaboration can help NGRNs adapt to their nursing roles [44, 75]. Therefore, interventions should focus not only on NGRNs but also on all nurses within the unit. For example, Chen et al. [44] suggested using role‐play or other intervention strategies to facilitate more effective collaboration among NGRNs, nurse managers, and senior nurses.

In addition, because individual resilience can be enhanced through training and because approaches such as mindfulness‐based or psychoeducational programs have been shown to effectively improve individuals’ resilience [76, 77], additional resilience‐enhancement interventions are recommended for NGRNs. Such interventions may further strengthen their resilience, thereby increasing their intention to stay and improving nurse retention.

### 5.3. Strengths and Limitations

This study has several limitations. **First**, since the duration of our study overlapped with the COVID‐19 outbreak, the impact of the pandemic or the hospital’s response mechanisms may have affected the work conditions faced by NGRNs and thus influenced the results of this research. Therefore, our findings may not fully represent the situation of NGRNs in the absence of the pandemic. **Second**, because this study used a self‐report questionnaire, participants may not have responded entirely in accordance with their actual circumstances. **Third**, owing to the lack of a control group, we cannot rule out the possibility that our findings reflect natural maturation among NGRNs regardless of their participation in the training program. **Fourth**, the distribution of work departments among the participants was uneven, with most participants coming from intensive care units, critical care units, and medical wards. Although we did not find these departments to have a significant effect on the outcome variables, they may have influenced our results. **Fifth**, the researchers in this study included administrators or staff members from the hospital where the participants were recruited, which may have introduced social desirability bias. **Sixth,** participants who withdrew from the study had a higher turnover rate and lower satisfaction with their work environment, resilience, and intention to stay, which may introduce selection bias and influence the study results.

One strength of our study lies in the fact that we conducted a longitudinal survey to investigate the effects of the two‐year on‐the‐job training program. Furthermore, on the basis of longitudinal data, we demonstrated the mediating effect of individual resilience on the relationship between satisfaction with the work environment and the intention to stay in this context.

## 6. Conclusion

Our study used a three‐wave survey to investigate the effects of a two‐year on‐the‐job training program on NGRNs. We observed that NGRNs’ satisfaction with their work environments and intention to stay exhibited a decreasing trend across the different waves of the survey, whereas their individual resilience exhibited an increasing trend. In addition, we observed a significant mediating effect, according to which individual resilience mediated the relationship between NGRNs’ satisfaction with their work environments and their intention to stay.

On the basis of these results, this two‐year on‐the‐job training program may significantly improve NGRNs’ individual resilience. However, given their satisfaction with the work environment and the intention to stay, this on‐the‐job training may need to be adjusted. Alternatively, additional measures may be needed to mitigate the effects of job changes on NGRNs.

## Funding

This study was supported by Taipei Tzu‐Chi Hospital (grant numbers: TCRD‐TPE‐110‐53 and TCRD‐TPE‐111‐55).

## Conflicts of Interest

The authors declare no conflicts of interest.

## Supporting Information

Additional supporting information can be found online in the Supporting Information section.

## Supporting information


**Supporting Information** Supporting Information 1: Content of the two‐year on‐the‐job training program.

## Data Availability

The data that support the findings of this study are available from the corresponding author upon reasonable request.
