# Supplementary material for: A Study of Changes in Newly Graduated Registered Nurses’ Resilience, Satisfaction With the Work Environment, and Intention to Stay During a Two‐Year Training Program and Corresponding Mediating Effects: A Longitudinal Study
Source: J Nurs Manag. 2026 May 10;2026:9633853. doi: 10.1155/jonm/9633853 (PMC13158279; doi:10.1155/jonm/9633853)
Supplement: Supplementary file 1 — Supporting Information Supporting Information 1: Content of the two‐year on‐the‐job training program. [file JONM-2026-9633853-s001.docx]

**Supplementary Material 1.**

**Content of the two-year on-the-job training program**

**1. Basic curriculum stage**

**(1) Orientation Training Course**

**Objectives:**

1. Understand the practice environment and regulatory framework
2. Possess knowledge and skills to maintain patient safety

**Training Content:**

1. **Hospital Introduction:** History, mission, vision, organizational structure and facility tour
2. **Nursing Department Introduction:** Nursing department mission and philosophy, administration, quality management, education and competency advancement system
3. **Operations Related to Nursing Personnel:** Personnel operations, employee benefits, fire safety, hospital information systems (including nursing information systems), etc.
4. **Nursing Work-Related Operations:**
   - Patient care models
   - Nursing documentation and common form’s introduction
   - Quality Management (I): Introduction to nursing quality concepts, adverse event prevention and reporting procedures, sharps injury prevention (employee safety)
   - Infection Control: Isolation measures and protective standards for special emerging infectious diseases (such as COVID-19), including standard, airborne, droplet, and contact precautions; demonstration and return demonstration of donning/doffing personal protective equipment; TOCC
   - Patient Safety Care: Patient identification, medication safety, blood transfusion safety, fall prevention, key points and principles of medical-nursing-patient handover
   - Trauma Emergency Care: Basic Life Support (BLS), including Cardio-Pulmonary Cerebral Resuscitation (CPCR) or Basic Traumatic Life Support (BTLS) techniques; Heimlich maneuver; Automated External Defibrillator (AED) training
   - Common Examination (or Laboratory) Procedures: Safety and precautions for specimen collection (blood, urine, feces)

**Training Time:** 5 days, all content must be completed within one month of employment

**Training Methods:** Lectures, video viewing, online or video learning, demonstration, return demonstration

**Assessment Standards:** Short-answer tests, self-assessment, checklists, teaching evaluations, etc.

**(2) New Employee Training Course Content**

**Objectives:**

1. Possess professional knowledge and skills required for patient care
2. Correctly and safely perform nursing techniques
3. Accept responsibility for patient care

**Training Content:**

1. **Professional Skills:**
   - Nursing care for common diseases, examinations and treatments in the unit
   - Actions and side effects of commonly used medications, medication and patient drug safety
   - Practice of common nursing techniques
   - Handling of common clinical health problems
   - Discharge preparation service concepts, case referral processes, case management care, etc.
   - Physical assessment skills, nursing information system operation
   - Ward routines: admission, consultation, bed transfer, discharge, hospital transfer, etc.
   - Operation of common medical equipment
   - Patient safety care-related courses and clinical practice
   - Geriatric care: Ethics and legal courses related to geriatric medicine; communication with elderly patients regarding mental health, adjustment, and their families; common disease patterns and syndromes in elderly patients
2. **Humanities Education:**
   - New employee role, stress management and adjustment (including stress coping for nursing staff during pandemics), spiritual care
   - Effective communication – A. Medical-nursing communication (including correct medical abbreviations); B. Nurse-patient communication
   - Employee rights: Labor Standards Act, Gender Equality in Employment Act, Sexual Harassment Prevention Act, medical violence handling, etc.
3. **Practical Operations:**
   - First month: Care for 6 to 8 patients together with preceptor
   - Second month: Independently care for 4 to 6 patients under preceptor guidance
   - Third month: Independently care for 6 to 8 patients under preceptor guidance

*Note: Use Ministry of Health and Welfare announcements as reference for patient care numbers, while appropriately adjusting based on trainee capability*

1. **Entrustable Professional Activity – Handover (within unit)**

*Note: The above recommended care numbers refer to day shifts; however, the standard for care numbers must still be adjusted by the preceptor, team leader, or nurse manager based on trainee capability to ensure patient safety.*

**Training Time:** 3 months

**Training Methods:** Clinical nursing practical learning, lectures, video viewing, online or video learning, demonstration, return demonstration

**Assessment Standards:**

1. Medication tests (written), new employee training manual or training item list, new employee training assessment
2. Training hospitals should provide appropriate assessment methods for individual training content

**2. Core Curriculum Stage**

**Year 1 Entry-Level Nurse Clinical Professional Competency Training**

**Objectives:**

1. Possess professional capability to care for general patients and be competent in clinical care work
2. Follow regulations and professional ethical standards to maintain patient safety and rights
3. Understand interdisciplinary team collaborative care concepts and cultivate collaborative care models

**Training Content:**

1. **Professional Skills:**
   - Nursing care for common diseases, examinations and treatments; general pain assessment and nursing
   - Actions and side effects of commonly used medications, medication and patient drug safety
   - Practice of common nursing techniques
   - Handling common clinical problems: Processing voluntary discharge procedures, unauthorized absence, complaint handling, etc.
   - Case Health Problem Analysis and Management (I): Application of nursing process
   - Quality Management (II): Introduction to nursing quality concepts, standard setting and monitoring, and new hospital accreditation quality management activities
   - Infection control training (as regulated by CDC, Ministry of Health and Welfare)
   - Patient Rights: Shared Decision Making (SDM), informed consent, Do Not Resuscitate (DNR), and Advance Decision (AD)
   - Patient safety promotion and case analysis
   - Suicide prevention and management
   - Understanding interdisciplinary team collaborative care (such as case managers, nurse practitioners, dietitians, physical or occupational therapists, and roles and tasks of various medical personnel)
   - Courses on preventive healthcare and health promotion for elderly patients
2. **Humanities Education:**
   - Nursing ethics and related legal issues: Introduction to Medical Care Act, Nursing Personnel Act, and nursing ethical dilemmas
   - Basic literacy: Career planning, stress adjustment and management (including mental health promotion and resilience for nursing staff during pandemics), caring
   - Hospice care concepts: Introduction to Hospice Palliative Care Act and hospice nursing concepts
3. **Self-Growth:**
   - Literature review
   - Book reports (or evidence-based nursing reports)
4. **Practical Operations:** Independently care for 7 to 11 patients under guidance of nurse manager, team leader, or preceptor (adjusted according to patient condition severity)
5. **Entrustable Professional Activity – Handover (between units)**

*Note: The above recommended care numbers primarily refer to day shifts; however, the standard for care numbers must still be adjusted by the preceptor, team leader, or nurse manager based on trainee capability to ensure patient safety.*

**Training Time:** 9 months

**Training Methods:** Clinical nursing practical learning, lectures, video viewing, online or video learning, demonstration, return demonstration, participation in interdisciplinary case discussion meetings

**Assessment Standards:** Training hospitals should provide specific and appropriate assessment methods for advancement personnel evaluation and individual training content

**3. Professional Course Stage**

**Year 2 Entry-Level Nurse Clinical Professional Competency Training**

**Objectives:**

1. Possess professional capability to care for critically ill patients
2. Follow regulations and professional ethical standards to maintain patient safety and rights
3. Understand interdisciplinary team collaborative care concepts and participate in collaborative care models

**Training Content:**

1. **Professional Skills:**
   - Nursing assessment: Including ECG, abnormal laboratory values, ABG collection and interpretation, tumor or chronic pain assessment (determined by hospital or unit characteristics)
   - Specialized nursing techniques (determined by hospital or unit characteristics)
   - Care for critically ill or difficult patients
   - Quality Management (III): Participate in establishing nursing standards, monitoring, and continuous quality improvement activities
   - Infection control training (as regulated by CDC, Ministry of Health and Welfare)
   - Organ donation handling procedures
   - Interdisciplinary team collaborative care training
   - According to practice unit, plan elderly patient-related care issues such as: polypharmacy, nutritional care, rehabilitation principles, cognitive-emotional and behavioral adjustment, etc.
   - According to practice unit, plan for medical personnel to receive Advanced Life Support (ALS), Advanced Cardiac Life Support (ACLS), Neonatal Resuscitation Program (NRP)/Advanced Pediatric Life Support (APLS), Emergency Trauma Training Course (ETTC) or Advanced Trauma Life Support (ATLS), post-traumatic psychological adjustment/psychological disaster prevention and other trauma emergency care training courses
2. **Humanities Education:**
   - Nursing ethics and related legal issues: Ethical issues (autonomy, informed consent, privacy, confidentiality), medical disputes, case discussions on emotional exhaustion and ethical dilemmas for nursing staff during pandemics, etc.
   - Communication (including listening skills), negotiation, conflict resolution skills and teamwork
   - Stress adjustment methods and practical applications
   - Understanding health insurance-related policies (such as: health insurance system, DRG, etc.) and medical institution response measures
3. **Self-Growth:**
   - Teaching and learning (health education techniques)
   - Case Health Problem Analysis and Management (II): Case analysis written report
4. **Practical Operations:** Independently care for 7 to 11 patients under guidance of nurse manager, team leader, or preceptor, according to patient condition (adjusted according to patient condition severity)
5. **Entrustable Professional Activity – Handover (interprofessional)**

*Note: The above recommended care numbers primarily refer to day shifts; however, the standard for care numbers must still be adjusted by the preceptor, team leader, or nurse manager based on trainee capability to ensure patient safety.*

**Training Time:** 12 months

**Training Methods:** Clinical nursing practical learning, internal and external lecture courses, video viewing, online or video learning, demonstration, return demonstration, participation in interdisciplinary case discussion meetings, exploring care cases using evidence-based nursing concepts

**Assessment Standards:** Training hospitals should provide appropriate assessment methods for advancement personnel evaluation and individual training content
